# Serum beta-2 microglobulin as a diagnostic biomarker for pediatric Epstein–Barr virus infections: a retrospective study

**DOI:** 10.3389/fped.2026.1853250

**Published:** 2026-06-30

**Authors:** Sheng Cheng, Jingjing Pan, Bing Wang, Xulong Cai, Tongjin Yin

**Affiliations:** Department of Pediatrics, Affiliated Hospital 6 of Nantong University, Yancheng Third People’s Hospital, Yancheng, Jiangsu, China

**Keywords:** beta-2 microglobulin, child, Epstein–Barr virus, primary infection, vitamin D3

## Abstract

**Objective:**

This study aims to evaluate serum beta-2 microglobulin (β2M) as a diagnostic biomarker for acute primary Epstein–Barr virus (EBV) infection in children, Additionally, serum vitamin D3 levels were compared between EBV-infected children and children with viral upper respiratory tract infection to explore their potential clinical relevance.

**Methods:**

A total of 129 children with acute primary EBV infection were identified as the EBV group, while 130 children with viral upper respiratory tract infections in the same age group hospitalized during the same period were identified as the control group. The age, gender, and laboratory indicators of the two groups were analyzed and compared. Pearson's correlation was used to analyze the correlation between β2M and serum EBV-DNA concentration. The receiver operating characteristic (ROC) curve was drawn, and the threshold and area under the curve (AUC) of β2M for predicting the diagnosis of EBV infection were calculated.

**Results:**

The white blood cell count, serum lactate dehydrogenase, and alanine aminotransferase levels in the EBV group were significantly than those in the control group (*P* < 0.0001), and Serum β2M level were significantly higher in the EBV group than in the control group (*P* < 0.001), while serum vitamin D3 levels were significantly lower (*P* < 0.001). Pearson's correlation analysis showed a positive correlation between serum β2M levels and serum EBV-DNA load (*r* = 0.469, *P* < 0.01). ROC curve analysis revealed that the AUC of serum β2M for diagnosing EBV infection was 0.907 (95% confidence interval 0.8723–0.9412; *P* < 0.01), with an optimal threshold of 3.3 mg/L, a specificity of 62.1%, and a sensitivity of 90.8%.

**Conclusion:**

The serum β2M levels are significantly increased in the stage of primary EBV infection in children, and β2M appears to be a promising biomarker for predicting primary EBV infection. The serum vitamin D3 levels are significantly reduced in children with primary EBV infection, and the protective effect of vitamin D supplementation in those children warrants additional clinical exploration.

## 1 Introduction

Epstein–Barr virus (EBV) infection is a common viral infection in children and is a lymphotropic herpesvirus. There are significant differences in the infection rates of children across various regions, with the peak of infection in China, for instance, occurring between 4 and 6 years of age (1, 2). Most children with primary EBV infection manifest as infectious mononucleosis (IM), and some children manifest as chronic active EBV (CAEBV) infection and EBV-related hemophagocytic lymphohistiocytosis (EBV-HLH) (3). The early clinical manifestations of EBV infection are principally manifested in fever, pharyngeal inflammation, and lymph node enlargement. It can be easily confused with pharyngeal tonsillitis caused by bacterial infection. The symptoms of infection in infants are often less typical (4). Some children may present with complications such as acute interstitial nephritis, cranial nerve palsy, encephalitis, hemolytic anemia, meningitis, myocarditis, thrombocytopenia, upper respiratory tract obstruction, and other complications that seriously threaten their health (5). Current management of pediatric EBV infection relies on supportive care for uncomplicated cases. In instances of severe manifestations, such as hemophagocytic lymphohistiocytosis, severe hepatitis, and airway obstruction, antiviral agents such as ganciclovir and short-term glucocorticoids may be utilized (3). However, specific therapies targeting early infection remain limited, and timely intervention is contingent upon accurate diagnosis—underscoring the clinical value of identifying reliable biomarkers. This diagnostic challenge is two-fold: delayed laboratory confirmation may prevent timely treatment, and earlier diagnosis could allow patients to benefit from interventions during the optimal therapeutic window. Clinically verified EBV infection is primarily diagnosed based on clinical symptoms, serum antibodies, and peripheral blood characteristics with certain difficulties and challenges arising in the diagnosis of atypical clinical symptoms (6). Therefore, in addition to specific antibodies and plasma EBV-DNA viral load, identifying biochemical markers of EBV infection can aid in clinical diagnosis.

Studies indicate that beta-2 microglobulin (β2M) is crucial for immune surveillance and regulation, with elevated levels observed in systemic inflammation and infection-induced autoimmune diseases (7). β2M is a low-molecular-weight protein present on the surface of nucleated cells, including lymphocytes and macrophages. Due to T and B cell activation, β2M is released, exceeding the reabsorption level of the renal tubules and increasing its serum concentration level (8). An increase in β2M level indicates an increase in cell turnover or a decrease in renal function. In various pathological states, β2M levels may increase significantly (9–11). The expression of vitamin D receptor (VDR) is primarily in B lymphocytes, which is also closely related to β2M expression (12). Vitamin D must bind to the VDR to exert various physiological effects. EBV infection is a lymphocytic disease, and the potential impact of this infection on β2M and vitamin D levels warrants further clinical investigation. This study aims to evaluate serum β2M as a diagnostic biomarker for acute EBV infection in children, and to compare serum β2M and vitamin D3 levels between EBV-infected children and children with viral upper respiratory tract infection, in order to support more timely and accurate diagnosis of pediatric primary EBV infection. To our knowledge, this is the first study to specifically evaluate serum β2M as a diagnostic biomarker for pediatric acute primary EBV infection. Although β2M has been investigated in other respiratory and systemic infections, its diagnostic value in the context of EBV infection in children has not been previously reported, which constitutes the novel contribution of this work.

## 2 Methods

### 2.1 Study population and design

This study used a retrospective cross-sectional design. All laboratory and clinical parameters were obtained at a single time point, corresponding to hospital admission prior to the initiation of specific therapy. The study included children aged 0–18 years, consistent with the standard definition of the pediatric age group. Children hospitalized with acute primary EBV infection admitted to the Sixth Affiliated Hospital of Nantong University between January 2021 and December 2022 were identified through the hospital's electronic medical record system and included in this retrospective analysis. Controls were children hospitalized for clinically and laboratory-confirmed viral upper respiratory tract infection during the same period, frequency-matched to cases by age and sex. Inclusion required: (1) clinical diagnosis of viral upper respiratory tract infection; (2) negative EBV serology (VCA-IgM, VCA-IgG, NA-IgG) and negative EBV-DNA PCR; and (3) absence of bacterial co-infection, chronic disease, or renal impairment. We acknowledge that this group represents a clinically relevant comparator with an active inflammatory process rather than healthy children (see Limitations). Data use for controls complied with the same institutional ethics policies as the study group, with informed consent waived.

The inclusion criteria for children with acute primary EBV infection were as follows: (1) Clinical symptoms: The course of the disease was ≤7 days and met at least three clinical criteria: fever, pharyngeal inflammation, cervical lymph node enlargement, hepatosplenomegaly, and eyelid edema. (2) Laboratory evidence: It was necessary to meet both serum EBV-DNA positive and any of the following serological test results: (1) Anti-EBV-CA-IgM positive with anti-EBV-CA-IgG and anti-EBV-NA-IgG, and (2) Anti-EBV-CA-IgM positive with anti-EBV-CA-IgG, but anti-EBV-NA-IgG negative.

Patients with chronic conditions such as malnutrition symptoms; diagnosed with congenital heart disease; having acute and chronic kidney disease; experiencing autoimmune diseases; suffering from chronic infectious illnesses, including tuberculosis and hepatitis B, were excluded.

### 2.2 Treatment course

Upon admission, patients were diagnosed with primary EBV infection. The therapy followed the principles outlined in the “Expert Consensus on Diagnosis and Treatment of Children's EB Virus Infection-Related Diseases” (3).

The components of the treatment protocol are as follows: (1) Hepatoprotection- glutathione 10–20 mg/kg/day IV and ursodeoxycholic acid 10–15 mg/kg/day oral for transaminase elevation; (2) Antiviral-ganciclovir 5 mg/kg per dose IV twice daily for 10–14 days in severe cases like hemophagocytic lymphohistiocytosis and rapidly progressive; (3) Immunomodulation-methylprednisolone 1–2 mg/kg/day IV for 3–5 days for upper respiratory obstruction; persistent fever>1 week; (4) Antibacterial-3rd generation cephalosporins like cefotaxime 50–100 mg/kg/day IV for confirmed bacteria coinfection.

Treatment is described for completeness; because all biomarkers were obtained from records at admission prior to therapy, no treatment-stratified biomarker comparisons were performed.

### 2.3 Clinical and laboratory parameters

Detailed records of demographic information (age, gender), clinical data (month of onset), and laboratory parameters were extracted from electronic medical records. All laboratory tests were retrieved from blood samples obtained at the time of hospital admission, prior to the initiation of specific antiviral therapy. The following laboratory parameters were assayed:Complete Blood Count (CBC): comprising White Blood Cell count (WBC), Hemoglobin (Hb), and Platelet count (PLT), quantified using an automated hematology analyzer (units: ×10^9^/L for WBC, g/L for Hb, ×10^9^/L for PLT). Biochemical Parameters: including C-Reactive Protein (CRP, determined by immunoturbidimetry, mg/L), Erythrocyte Sedimentation Rate (ESR, measured via the Westergren method, mm/h), Lactate Dehydrogenase (LDH, assessed by enzymatic assay, U/L), Alanine Aminotransferase (ALT, measured enzymatically, U/L), Blood Urea Nitrogen (BUN, determined by enzymatic method, mmol/L), Serum Ferritin (measured by chemiluminescence immunoassay, μg/L), and Serum β2-microglobulin (β2M, quantified by immunoturbidimetry, mg/L). Immunological/Virological Parameters: encompassing Vitamin D3 (measured by chemiluminescence immunoassay, ng/mL), Serum EBV-DNA load (detected by real-time quantitative PCR, copies/mL), and EBV antibodies (EBV-CA-IgM, EBV-CA-IgG, EBV-NA-IgG, analyzed using chemiluminescence or ELISA). D-dimer (D-D) level was also quantified (measured by immunoturbidimetry, μg/L).

All data, including demographic information, routine clinical laboratory parameters, and specific investigations such as EBV PCR, EBV antibodies (EBV-CA-IgM, IgG, EBV-NA-IgG), β2M, and vitamin D3, were retrieved from electronic medical records of hospitalized patients as part of their standard clinical care for diagnostic and management purposes. For the control group, similar routine clinical laboratory parameters and diagnostic tests for common viral infections were retrieved retrospectively from their medical records. To ensure comparability, β2M and vitamin D3 levels were also available in the control group from samples that had been drawn during their hospitalization, provided these tests were part of their clinical workup for the viral upper respiratory tract infection. In our institution, serum β2M is routinely measured as part of the standard renal function panel for all hospitalized children prior to medication administration. Therefore, β2M values for both the EBV and control groups were retrospectively retrieved from the existing laboratory records, rather than being measured specifically for this study.

### 2.4 Statistical analysis

Prism statistical software (version 10.1) was used for the statistical analysis. The normality of continuous variables was assessed using the Shapiro–Wilk test. As most continuous variables were non-normally distributed, all continuous variables are reported as median (interquartile range, IQR) and were compared between groups using the Mann–Whitney *U* test to ensure methodological consistency. Categorical variables were compared using the Chi-square test. For the correlation analysis, EBV-DNA load was log_10_-transformed, and the association between serum β2M and EBV-DNA load was assessed using Pearson correlation. A two-sided *P* < 0.05 was considered statistically significant. Receiver operating characteristic (ROC) analysis was used to evaluate the diagnostic performance of the serum β2M vitamin D3, ALT, LDH, and WBC levels, providing area under the curve (AUC), sensitivity, and specificity values.

### 2.5 Ethical statements and subjects

This retrospective study utilized de-identified data from medical records. The Ethics Committee of Yancheng Third People's Hospital approved the study (Approval No.: 2021-O53-01) and waived the requirement for informed consent, in accordance with institutional guidelines for retrospective data analysis. The research plan was registered with the China Clinical Trial Registration Center (Registration No.: ChiCTR2200066383). The data of children with viral infections admitted from January 2021 to December 2022 were extracted from the electronic database and patient files of the Sixth Affiliated Hospital of Nantong University.

## 3 Results

### 3.1 Clinical and laboratory parameter results

**Figure 1:**
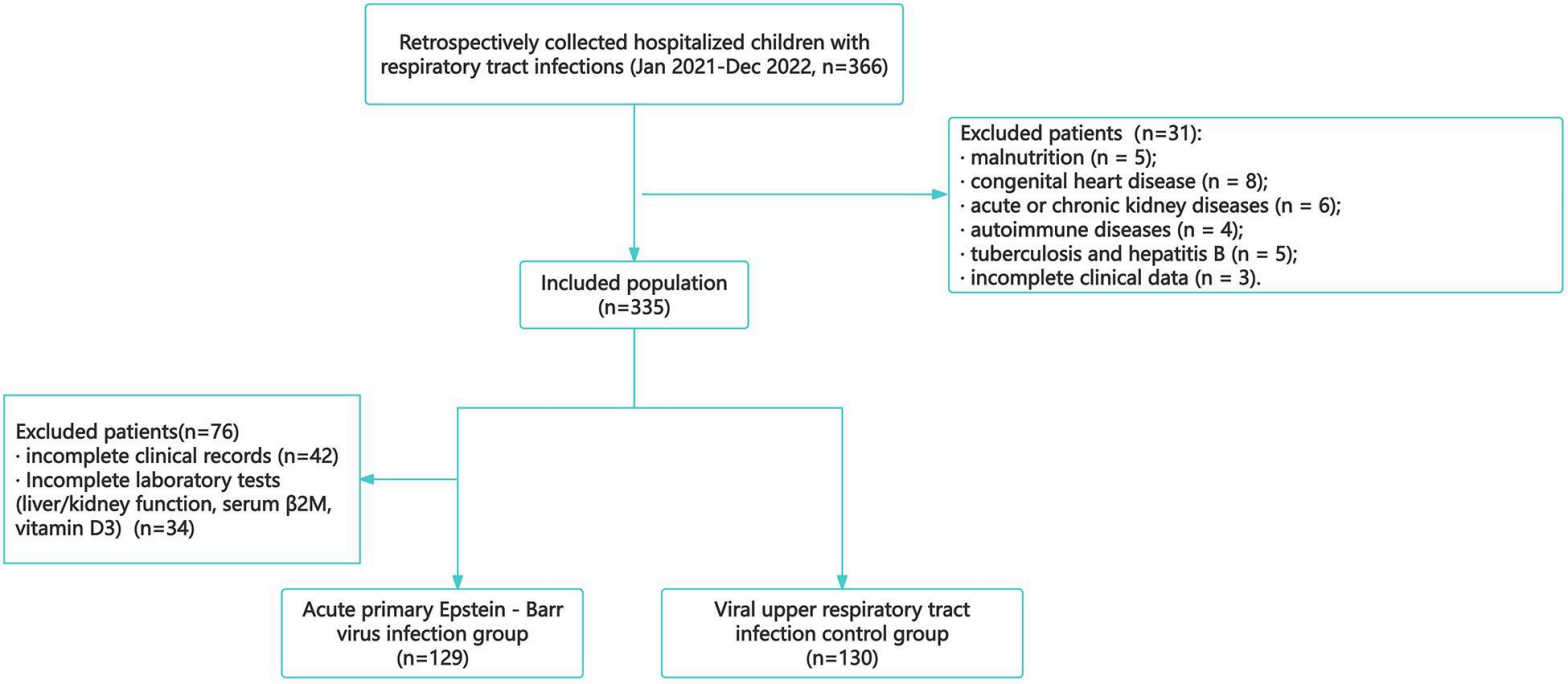
Flow chart for patient identification and inclusion in retrospective analysis. A total of 366 hospitalized children with respiratory tract infections (January 2021–December 2022) were retrospectively screened. After excluding 31 patients with chronic conditions or incomplete data, 335 children were eligible. Among these, 76 were further excluded due to incomplete clinical records or missing laboratory tests (liver/kidney function, serum β2M, vitamin D3). The remaining 259 children were divided into two groups: 129 with acute primary EBV infection (study group) and 130 with viral upper respiratory tract infection (control group). EBV, Epstein–Barr virus; β2M, beta-2 microglobulin.

The EBV group included 129 patients, whereas the control group comprised 130 patients (Figure 1). As shown in Table 1, patients in the EBV group had significantly higher levels of WBC (median 12.14 vs. 9.67 × 10^9^/L, *Z* = −4.02, *r* = 0.250, *P* < 0.0001), LDH (median 617.0 vs. 330.9 U/L, *Z* = −13.04, *r* = 0.810, *P* < 0.0001), and ALT (median 63.1 vs. 34.1 U/L, *Z* = −7.22, *r* = 0.449, *P* < 0.0001) compared to the control group. No other clinical or laboratory parameters showed statistically significant differences (Table 1).

**Table 1:** Clinical and laboratory parameters of children with acute primary EBV infection and viral upper respiratory tract infection controls.

Clinical indicators	Acute EBV infection group (*n* = 129)	Control group (*n* = 130)	Statistics (*Z*/*χ*^2^)	Effect size (*r*/*V*)	*P* value
Age (years)	7.5 (5.8–9.3)	7.1 (6.1–7.9)	−1.82	0.113	0.069
Gender (male), *n* (%)	70 (54.4)	69 (53.5)	0.04^a^	0.012^b^	0.837
WBC (×10^9^/L)	12.14 (8.37–15.19)	9.67 (8.68–11.20)	−4.02	0.250	<0.0001
Hb (g/L)	119 (112–127)	121 (115–128)	−1.10	0.068	0.274
PLT (×10^9^/L)	223.0 (182–276)	242 (163.7–334.2)	−1.24	0.077	0.213
CRP (mg/L)	6.51 (2.74–15.62)	5.81 (3.34–9.55)	−1.32	0.082	0.184
ESR (mm/h)	10 (5–16)	9 (4–19)	−0.28	0.017	0.773
D-Dimer (μg/L)	111.2 (65.5–186.5)	128.1 (87.5–168.3)	−1.29	0.080	0.198
LDH (U/L)	617.0 (416.3–795.4)	330.9 (283.9–372.2)	−13.04	0.810	<0.0001
ALT (U/L)	63.1 (37.6–91.4)	34.1 (27.7–44.0)	−7.22	0.449	<0.0001
Ferritin (μg/L)	167.9 (134.5–189.9)	155.4 (126.9–179.0)	−1.69	0.105	0.091
BUN (mmol/L)	3.59 (3.4–5.7)	3.81 (3.3–6.7)	−0.61	0.038	0.538

Data are median (interquartile range, IQR) expressed as (Q1–Q3) unless otherwise indicated. WBC, white blood cell count; Hb, hemoglobin; PLT, platelet count; CRP, C-reactive protein; ESR, erythrocyte sedimentation rate; D-Dimer; LDH, lactate dehydrogenase; ALT, alanine aminotransferase; BUN, blood urea nitrogen.

a *χ*^2^ value from Chi-square test.

b Effect size for continuous variables: rank-biserial correlation *r* = |*Z*|/√*N* (*N* = 259); for sex: Cramér's *V*.

### 3.2 Comparison of serum β2M and vitamin D3 levels between groups

**Figure 2:**
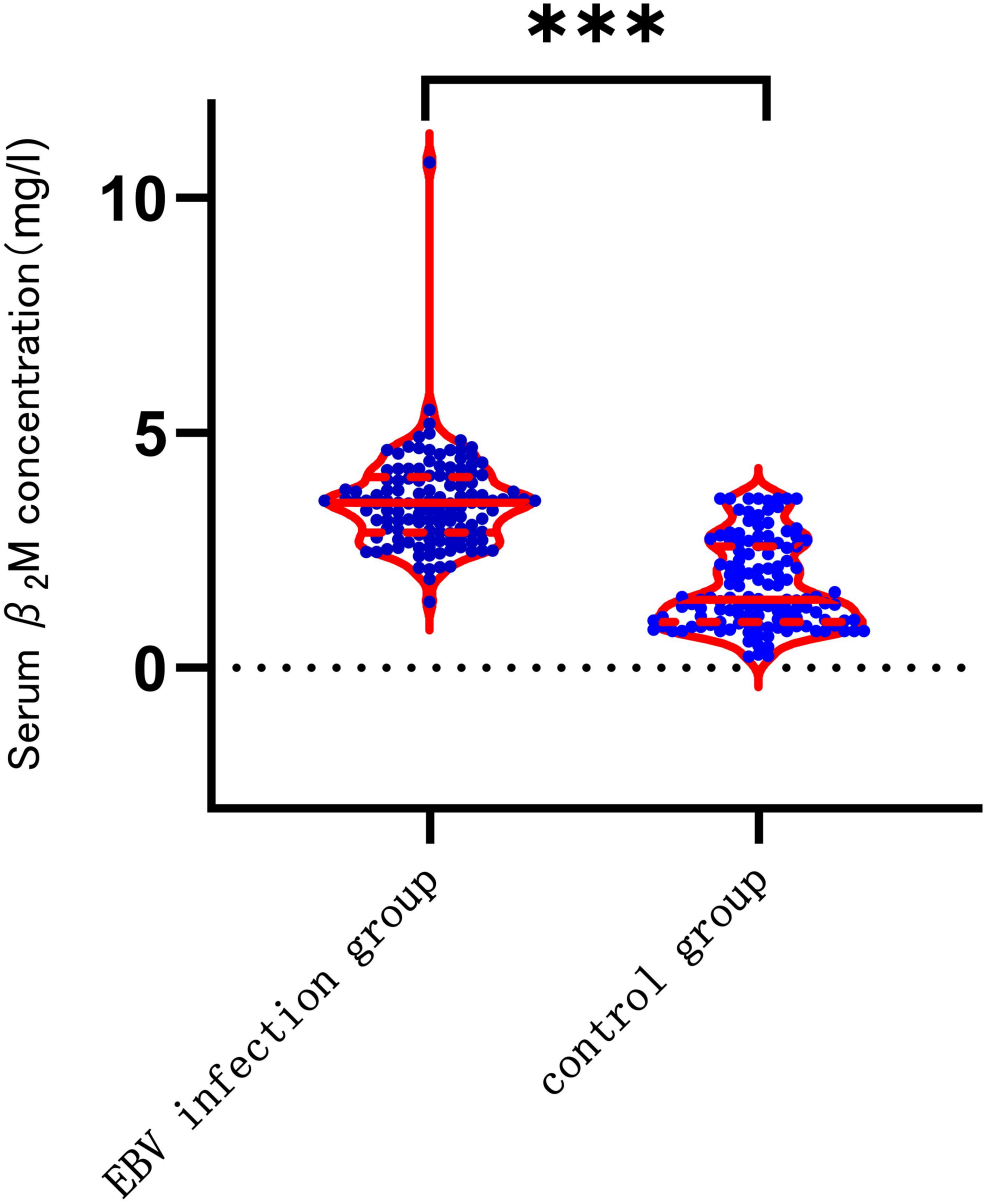
Comparison of serum β2M concentrations between the EBV group and the control group. Data are presented as median (interquartile range, IQR); whiskers indicate the 5th–95th percentiles. Statistical significance was determined using the Mann–Whitney *U* test. ***P < 0.001 vs. control group (β2M, beta-2 microglobulin).

**Figure 3:**
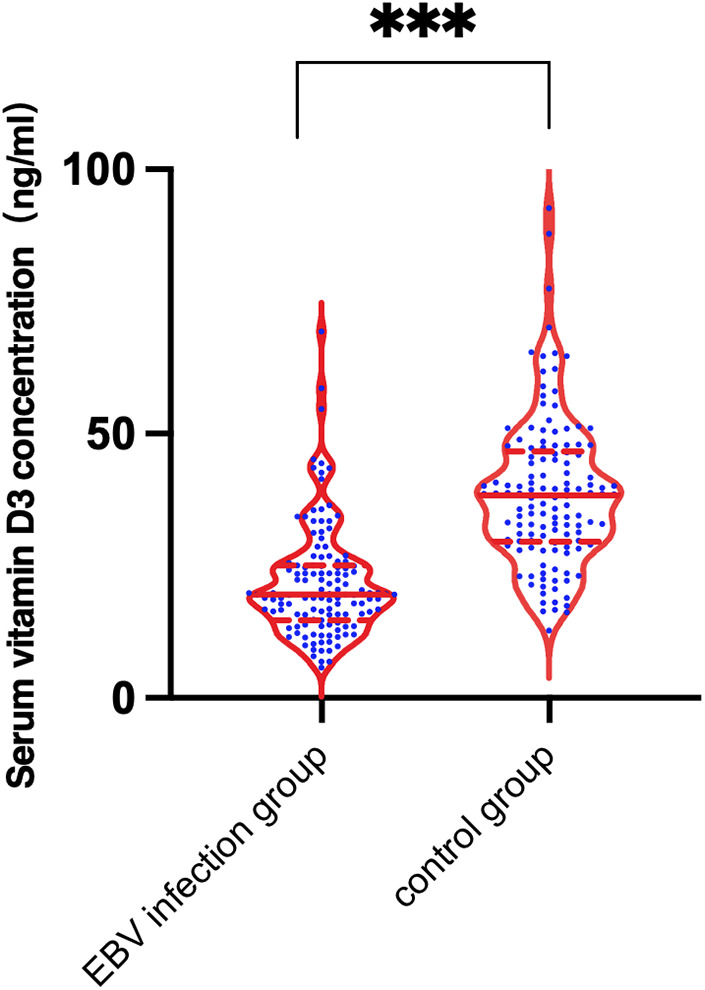
Comparison of serum vitamin D3 concentrationbetween EBV group and control group. Data are presented as median (IQR); whiskers indicate the 5th–95th percentiles. Statistical significance was determined using the Mann–Whitney *U* test. ***P < 0.001 vs. control group.

The serum β2M level (mg/L) in the EBV group was significantly higher than that in the control group [3.51 (2.87, 4.05) vs. 1.45 (0.97, 2.59)], with a statistically significant difference (*Z* = –11.28, *P* < 0.001). The serum vitamin D3 concentration (ng/mL) in the EBV group was lower than that in the control group [19.6 (14.8, 25.1) vs. 38.4 (29.5, 46.7)], with a statistically significant difference (*Z* = 10.47, *P* < 0.001; Figures 2, 3). Among the 129 EBV-infected children, 47 (36.4%) were negative for VCA-IgM and VCA-IgG at presentation but were confirmed by positive EBV-DNA; serum β2M was also elevated in this subgroup; this observation is descriptive only, as quantitative β2M records were incomplete for formal statistical comparison (see Limitations).

### 3.3 Comparison of the correlation between serum β2M level and serum EBV-DNA load in children with primary EBV infection

**Figure 4:**
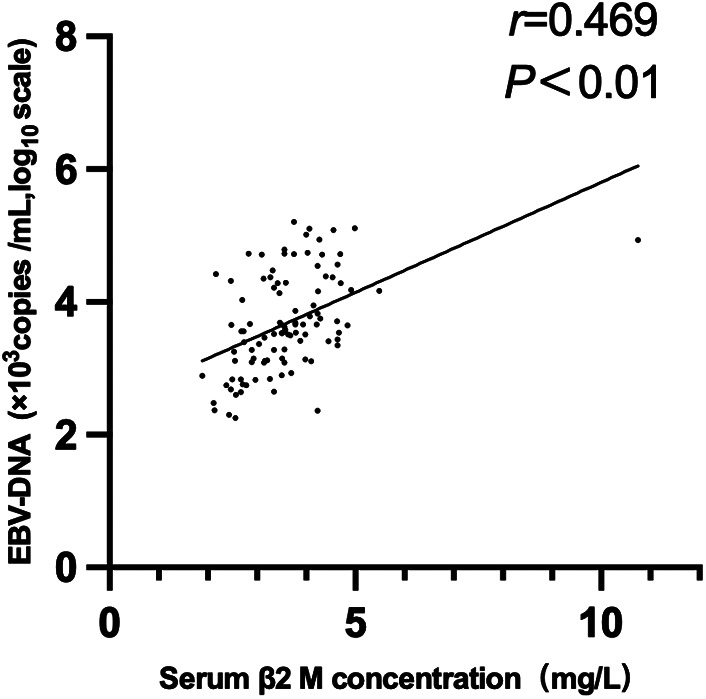
Correlation between serum β2M concentration and EBV-DNA load in children with acute primary EBV infection. Scatter plot showing the relationship between serum β2M (mg/L) and EBV-DNA load (×10^3^ copies/mL, log_10_-transformed). Pearson correlation analysis on log_10_-transformed data revealed a moderate positive correlation (*r* = 0.469, *P* < 0.01). The solid line represents the linear regression fit (β2M, beta-2 microglobulin).

Pearson's correlation analysis indicated that the β2M level (mg/L) in children infected with EBV was positively correlated with serum EBV-DNA (×10^3^ copies/mL; *r* = 0.469, *P* < 0.01; Figure 4).

### 3.4 Receiver operating characteristic (ROC) analysis of single biomarkers for predicting EBV infection

**Figure 5:**
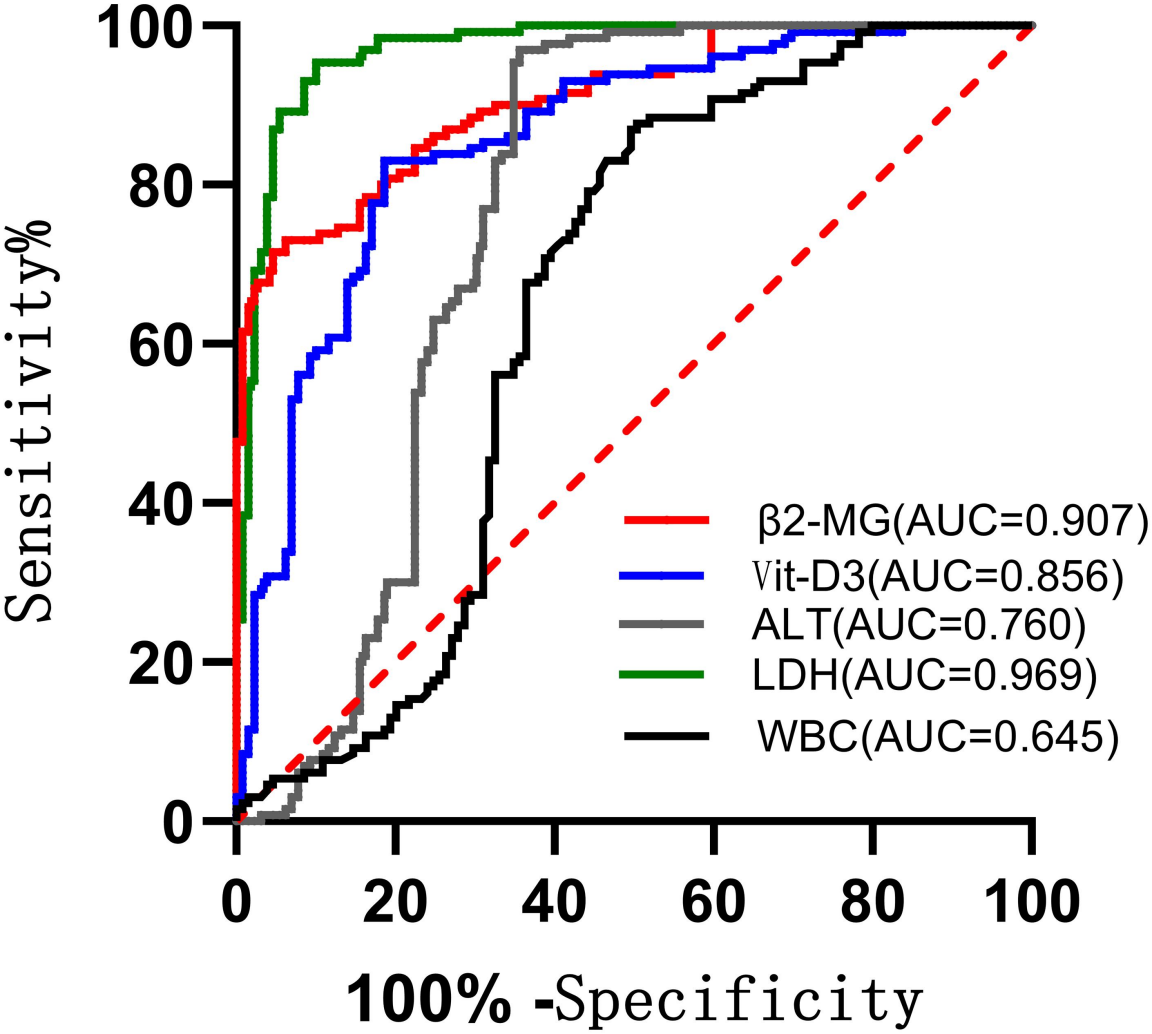
Receiver operating characteristic (ROC) curves of single biomarkers for predicting acute primary EBV infection in children. ROC curves are shown for serum β2M, vitamin D3, ALT, LDH, and WBC. The area under the curve (AUC) values are: LDH 0.969, β2M 0.907, vitamin D3 0.856, ALT 0.760, WBC 0.645. The diagonal dashed line represents the reference (AUC = 0.5).

ROC curves were generated for serum β2M, vitamin D3, ALT, LDH, and WBC to evaluate their individual diagnostic performance for EBV infection (Figure 5). The area under the curve (AUC) values were: LDH 0.969, β2M 0.907, vitamin D3 0.856, ALT 0.760, and WBC 0.645. Serum β2M exhibited a sensitivity of 90.8% and a specificity of 62.1% at the optimal cutoff of 3.3 mg/L.

## 4 Discussion

In this retrospective study, children with acute primary EBV infection exhibited significantly elevated serum β2M levels compared to those with viral upper respiratory tract infections. Furthermore, serum β2Mlevels showed a positive correlation with EBV-DNA concentration. Receiver operating characteristic (ROC) curve analysis demonstrated its high diagnostic efficacy for EBV infection, indicating that this indicator could serve as a potential biomarker for the diagnosis of primary EBV infection in children. Notably, serum vitamin D3 levels were significantly decreased in the EBV-infected group, which highlights the necessity of further investigating the protective effects of vitamin D supplementation in these pediatric patients.

β2M is a small-molecular-weight globulin encoded by human chromosome 15. Its polypeptide chain consists of 99 amino-acid residues. The tertiary structure of this protein exhibits a typical β-sandwich folding pattern, a structural feature that is highly conserved in immunoglobulin superfamily molecules (13). β2M is extensively expressed on the surface of all nucleated cells, especially in immune-active cells, including lymphocytes and monocytes (14). During antigen presentation, β2M forms a non-covalent complex with the heavy chain α domain of the main histocompatibility complex class I molecule (MHC-I), which has an important regulatory effect on antigen recognition by CD8+ cytotoxic T lymphocytes (13). Clinically, β2M has been established as a classic biomarker for evaluating glomerular filtration and tubular reabsorption function because of its biological properties in glomerular filtration and tubular metabolism (15). It has also demonstrated significant biomarker relevance in the pathological mechanisms of various non-renal diseases, including malignant tumors, autoimmune diseases, and chronic inflammatory conditions (16–18).

As a novel biomarker associated with inflammation, β2M is crucial in immune surveillance and regulation and has significantly increased the synthesis of systemic inflammation and autoimmune diseases caused by infection, including systemic lupus erythematosus, Crohn's disease, ulcerative colitis, and S-Jun's syndrome (7). We have previously shown that serum β2M levels differ between viral and bacterial lower respiratory tract infections in children and correlate with specific viral subtypes (19). In this study, we observed that the average β2M level of children with acute EBV infection was significantly higher than that of children with viral infection in the control group; among the 129 children with EBV infection, 47 children had VCAIgM/VCAIgG negative 36.4%, which was confirmed by positive EBV-DNA testing at admission. Therefore, an increase in β2M levels can be a clue to EBV infection, especially in the early stages of EBV infection. Previous research indicates that β2M concentration reflects the degree of active lymphocyte proliferation (20), with EBV infection being the main reason for the active proliferation of lymphocytes (21). Consequently, we believe that the increase in β2M is due to the excessive proliferation of EBV-infected lymphocytes, and β2M expression also increases, exceeding the limit of reabsorption of normal renal tubules, thus leading to an increase in serum β2M (22, 23).

Regarding diagnosis, the identification of pediatric EBV infection predominantly depends on the detection of viral antibodies, nucleic acid testing, and various other methodologies. Nevertheless, diagnostic challenges remain in certain cases, particularly during the early stages of infection or when atypical clinical manifestations are present. Children with EBV infection often exhibit typical clinical “triad symptoms, including pharyngeal inflammation, exudation, and lymph node enlargement,” but others only have some symptoms. Studies have revealed that the specificity and sensitivity of typical “triad symptoms” associated with EBV infection are 68.2% and 41.9%, respectively (24). Consequently, it poses certain difficulties in early clinical diagnosis. PCR detection of EBV-DNA is an important indicator for the pathogenic diagnosis of EBV and is of great value in the diagnosis of primary EBV infection (25). In this study, the correlation analysis of β2M levels and EBV load in children with EBV infection suggested a significant positive correlation. Accordingly, β2M can be used as a biochemical index for promising EBV infections. This significantly increased serum β2M levels in children with fever, suggesting a possible EBV infection, providing clinically important clues for the diagnosis of EBV infection, and can also be used as a predictive biochemical index for EBV infection. In the ROC analysis, LDH exhibited the highest AUC of 0.969 among the evaluated single markers. However, it is important to note that elevated LDH levels are a non-specific indicator of cellular damage and inflammation, which can occur in various infectious and non-infectious conditions. In contrast, although β2M had a lower AUC of 0.907, it demonstrated characteristics more relevant to EBV infection. The ROC curve analysis revealed that the optimal cutoff for β2M in predicting EBV infection in children was ≥3.3 mg/L, with a sensitivity of 90.8% and a specificity of 62.1%. Moreover, the correlation between serum β2M and EBV-DNA load was moderate (*r* = 0.469), suggesting that β2M elevation reflects not only viral replication but also concurrent lymphocyte activation, reinforcing its role as a general immune response marker rather than a specific surrogate for viral load. Consequently, serum β2M can serve as a ideal screening biomarker for EBV infection. As a serum biochemical marker, it offers reliable detection and cost-effectiveness. β2M levels were significantly elevated in children with clinically suspected EBV infection, and further assessment of EBV-specific antibodies and viral DNA can aid in clinical confirmation. However, since β2M is also elevated in other inflammatory and infectious conditions—including the viral upper respiratory infections observed in our control group—it should not be considered a stand-alone confirmatory test for EBV. Instead, in resource-limited settings where EBV serology and PCR are not readily available, a markedly elevated serum β2M in a febrile child with compatible clinical features may serve as a cost-effective triage signal, prompting targeted confirmatory testing or referral. Definitive recommendations will require prospective validation against healthy individuals and non-EBV infectious comparators, along with the evaluation of multi-marker panels and cost-effectiveness.

This study revealed a significant decrease in serum vitamin D3 levels among children with EBV infection. The average vitamin D3 level in 129 children was 21.7 ng/mL, and the median was 19.6 ng/mL. Compared with the control group during the same period, there was a significant decrease in vitamin D levels. These children did not have the epidemiological factors of vitamin D deficiency compared with the control group, including insufficient dietary sources and reduced sunlight exposure in households. As a result, the reduction of their levels has certain clinical significance. The reason for the decrease in serum vitamin D3 levels in children with EBV infection remains unclear. The author speculates that EBV infection is the disease that can cause B cell proliferation (26), and the expression of VDR is closely related to B cells. VDR is expressed by human peripheral B cells (12). B cell proliferation increases VDR expression, and vitamin D in the serum combines with VDR, leading to a decrease in vitamin D3 levels. However, it is unclear whether the reduction in sunshine exposure and dietary factors are affected by the disease, resulting in a decrease in vitamin D3 levels. Vitamin D enhances the innate immune response, promotes self-tolerance, and inhibits Th1 cell proliferation (27). It has been reported that VD can restore the fusion of viral-inhibited autophagy through VDR receptors, reduce cell apoptosis through VDR signaling pathways, and inhibit the expression of proinflammatory factors (28, 29). Further clinical research is necessary to determine whether vitamin D supplementation can play a therapeutic role in EBV-infected children or inhibit the excessive proliferation of EBV-infected B cells.

Regarding the potential association between β2M and disease severity, clinical outcomes of EBV infection in children exhibit a broad spectrum, ranging from mild symptoms to severe complications such as hemophagocytic lymphohistiocytosis (HLH). Although our current retrospective design did not include formal severity stratification or outcome-based subgroup analysis, emerging evidence from the literature suggests that elevated β2M levels may correlate with viral replication activity and the severity of immune impairment (30). Future prospective studies should incorporate standardized severity scoring to validate whether serum β2M can effectively stratify risk in pediatric EBV infection.

In managing pediatric EBV infections, current treatment strategies primarily focus on providing symptomatic relief (3). In more severe cases, the use of antiviral agents or immunomodulators may be indicated. Variations in β2M and vitamin D3 levels can provide valuable insights for tailoring treatment strategies. For instance, in future longitudinal studies, a markedly elevated β2M level accompanied by significantly low vitamin D3 level might indicate a more pronounced immune imbalance. In such cases, it may be prudent to implement more intensive immunomodulatory treatments along with appropriate vitamin D3 supplementation to enhance immune efficacy and facilitate recovery (29). Furthermore, prospective serial monitoring of these biomarkers during treatment may help evaluate therapeutic response and warrants investigation in future studies.

### 4.1 Limitations and future direction

This study has several limitations. First, the single-center retrospective design may introduce selection bias. Second, all biomarkers were measured only at admission; the absence of longitudinal follow-up precludes assessment of temporal β2M dynamics. Third, the control group comprised children with viral upper respiratory tract infections rather than healthy children; because such infections themselves can elevate β2M, the true difference between EBV infection and the healthy state may be even greater, but a healthy reference group is needed for definitive interpretation. Fourth, baseline vitamin D3 data were unavailable, preventing differentiation between pre-existing and infection-induced reductions. Fifth, severity- and treatment-stratified analyses were not feasible. Sixth, due to the retrospective design and incomplete quantitative records for the seronegative subgroup, we were unable to perform formal statistical comparisons of β2M levels between seronegative and seropositive cases. Future prospective, multicenter studies should include healthy controls, serial biomarker measurements, standardized severity scoring.

## 5 Conclusion

The serum β2M level of children with primary EBV infection increased significantly in the early stages of primary EBV infection, suggesting its potential as a diagnostic biomarker. Serum vitamin D levels were significantly reduced in children with primary EBV infection. The protective effect of vitamin D supplementation on children with EBV infection requires additional clinical evaluation.

## Data Availability

The raw data supporting the conclusions of this article will be made available by the authors, without undue reservation.
