# Supplementary material for: Serum beta-2 microglobulin as a diagnostic biomarker for pediatric Epstein–Barr virus infections: a retrospective study
Source: Front Pediatr. 2026 Jun 30;14:1853250. doi: 10.3389/fped.2026.1853250 (PMC13365129; doi:10.3389/fped.2026.1853250)
Supplement: Supplementary file 1 [file Datasheet1.pdf]

Supplementary Material – Routine Renal Function Panel at Our Institution

Purpose: This document is provided as supporting evidence to demonstrate that serum β2-microglobulin (β2M) is a routine component of the standard renal function panel at Yancheng Third People's Hospital, which is ordered for all hospitalized children as part of their baseline clinical assessment prior to medication administration.
Content: The attached document is an anonymized temporary medical order sheet from a child with pneumonia who was hospitalized at our institution in November 2022. This patient is not part of the study cohort.
Key information highlighted:
Date: November 25, 2022
Department: Pediatrics
Order: Renal Function Panel, 5 items
Purpose of order: Routine admission laboratory workup prior to medication
The Renal Function Panel in our institution includes serum β2-microglobulin along with other routine renal function markers. This confirms that β2M results are routinely available in the medical records of hospitalized children at our institution, and were therefore retrospectively retrievable for both the EBV group and the control group in this study.
Patient information: All patient-identifiable information (name, medical record number, bed number) has been redacted to ensure anonymity. This patient was not included in the present study.
Date of document: November 25, 2022 (within the study period of January 2021 – December 2022)

盐城市第三人民医院

临 时 医 嘱 单

姓名:[redacted] 科别:儿科 病区:南院三十二病区 床号:[redacted] 住院号:[redacted]

日期	时间	医嘱	医生	审核者	执行时间	执行护士
22-11-25	15:32	尿液分析(含尿沉渣定量)	[redacted]	[redacted]	22-11-25 15:55	[redacted]
22-11-25	15:32	粪便常规(病区)	[redacted]	[redacted]	22-11-25 15:55	[redacted]
22-11-25	15:32	肝功能10项	[redacted]	[redacted]	22-11-25 15:55	[redacted]
22-11-25	15:32	肾功能5项	[redacted]	[redacted]	22-11-25 15:55	[redacted]
22-11-25	15:32	电解质	[redacted]	[redacted]	22-11-25 15:55	[redacted]
22-11-25	15:32	红细胞沉降率测定(ESR)	[redacted]	[redacted]	22-11-25 15:55	[redacted]
22-11-25	15:32	降钙素原检测(PCT)	[redacted]	[redacted]	22-11-25 15:55	[redacted]
22-11-25	15:32	急诊心肌酶谱	[redacted]	[redacted]	22-11-25 15:55	[redacted]
22-11-25	15:32	肺炎支原体IgM抗体	[redacted]	[redacted]	22-11-25 15:55	[redacted]
22-11-25	15:32	呼吸道7种病毒抗原测定	[redacted]	[redacted]	22-11-25 15:55	[redacted]
22-11-25	15:32	细菌培养及鉴定(痰液)	[redacted]	[redacted]	22-11-25 15:55	[redacted]
22-11-25	15:32	总IgE测定	[redacted]	[redacted]	22-11-25 15:55	[redacted]
22-11-25	15:32	补体免疫球蛋白	[redacted]	[redacted]	22-11-25 15:55	[redacted]
22-11-25	15:32	血培养及鉴定(右侧需氧)	[redacted]	[redacted]	22-11-25 15:55	[redacted]
22-11-25	15:32	铁蛋白测定(SF)	[redacted]	[redacted]	22-11-25 15:55	[redacted]
22-11-25	15:32	血浆D-二聚体测定(仪器法)	[redacted]	[redacted]	22-11-25 15:55	[redacted]
22-11-25	15:32	甲型/乙型流感通用引物核酸检测	[redacted]	[redacted]	22-11-25 15:55	[redacted]
22-11-25	15:32	常规心电图(心电图室)	[redacted]	[redacted]	22-11-25 15:55	[redacted]
22-11-25	15:35	肺炎支原体/衣原体核酸检测	[redacted]	[redacted]	22-11-25 15:55	[redacted]
22-11-25	18:33	布洛芬混悬液 10ml Po	[redacted]	[redacted]	22-11-25 18:35	[redacted]
22-11-26	10:30	布洛芬混悬液 8ml Po	[redacted]	[redacted]	22-11-26 10:30	[redacted]
22-11-27	08:27	肺力咳合剂 15ml Po	[redacted]	[redacted]	22-11-27 08:45	[redacted]
22-11-29	08:34	急诊血液分析	[redacted]	[redacted]	22-11-29 09:31	[redacted]
22-11-29	08:34	肝功能10项	[redacted]	[redacted]	22-11-29 09:31	[redacted]
22-11-29	08:34	肺炎支原体IgM抗体	[redacted]	[redacted]	22-11-29 09:31	[redacted]
22-11-30	08:45	5%葡萄糖注射液 50ml i.v.gtt (续)	[redacted]	[redacted]	22-11-30 08:49	[redacted]
		注射用甲泼尼龙琥珀酸钠(冻干) 25mg				
22-12-01	09:38	出院	[redacted]	[redacted]	22-12-01 09:43	[redacted]

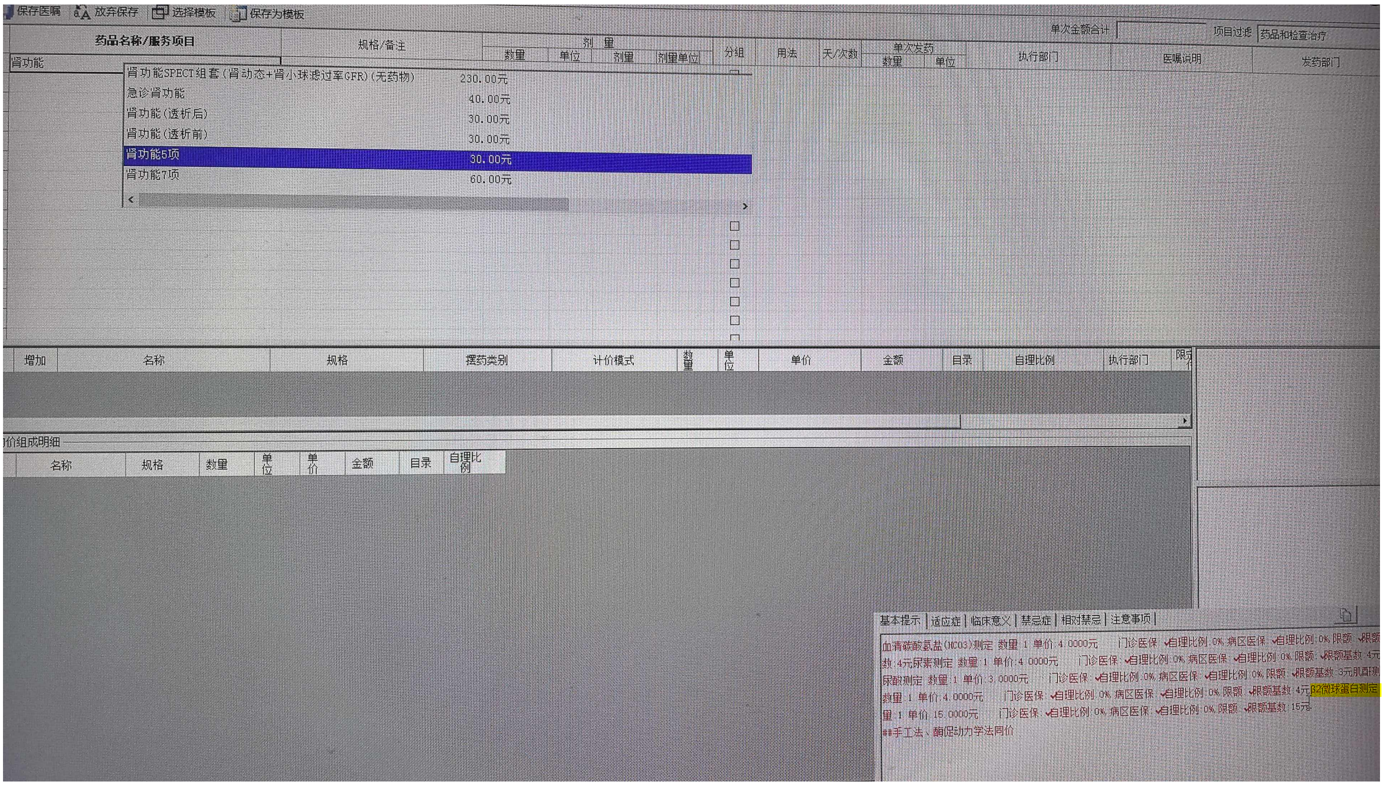

保存医嘱 放弃保存 选择模板 保存为模板
单次金额合计
项目过滤 药品和检查治疗
药品名称/服务项目
规格/备注
剂 量
数量 单位 剂量 剂量单位
分组
用法
天/次数
单次发药
数量 单位
执行部门
医嘱说明
发药部门
肾功能
肾功能SPECT组套(肾动态+肾小球滤过率GFR)(无药物) 230.00元
急诊肾功能 40.00元
肾功能(透析后) 30.00元
肾功能(透析前) 30.00元
肾功能5项 30.00元
肾功能7项 60.00元
增加 名称 规格 摆药类别 计价模式 数量 单位 单价 金额 目录 自理比例 执行部门
价组成明细
名称 规格 数量 单位 单价 金额 目录 自理比例
基本提示 适应症 临床意义 禁忌症 相对禁忌 注意事项
血清碳酸氢盐(HCO3)测定 数量 1 单价 4.0000元 门诊医保 ✓自理比例 0% 病区医保 ✓自理比例 0% 限额 ✓限额
数 4元尿素测定 数量 1 单价 4.0000元 门诊医保 ✓自理比例 0% 病区医保 ✓自理比例 0% 限额 ✓限额基数 4元
尿酸测定 数量 1 单价 3.0000元 门诊医保 ✓自理比例 0% 病区医保 ✓自理比例 0% 限额 ✓限额基数 3元肌酐测
数量 1 单价 4.0000元 门诊医保 ✓自理比例 0% 病区医保 ✓自理比例 0% 限额 ✓限额基数 4元β2微球蛋白测定
量 1 单价 15.0000元 门诊医保 ✓自理比例 0% 病区医保 ✓自理比例 0% 限额 ✓限额基数 15元
##手工法、酶促动力学法同价

盐城市第三人民医院

检验报告单

第1页\共1页

病人种类: **住院** 姓名[redacted] 性别[redacted] 年龄[redacted] 床号[redacted] 病人编号[redacted]

科室: 儿科 临床诊断: 支气管肺[redacted]

[redacted] **检验项目: 补体免疫球蛋白，电解质，肝功能10项，降钙素[redacted]），肾功能Ⅱ**

[redacted]静脉血 标本收到时间: 11-25 16:58 [redacted]

名 称	结果	参考范围	单位	名 称	结果	参考范围	单位
总胆红素*	4.5	≤23	μmol/L	钙*	2.20	2.1--2.8	mmol/L
直接胆红素*	<1.5	≤8	μmol/L	磷*	1.38	1.25--1.93	mmol/L
总蛋白*	67.4	65--84	g/L	镁*	1.08 ↑	0.75--1.02	mmol/L
白蛋白*	43.9	39--54	g/L	铁*	5.15 ↓	7.8--32.2	μmol/L
球蛋白	23.5	18--38	g/L	二氧化碳结合力	20.1	20--31	mmol/L
白球比	1.87	1.2--2.5		尿素*	4.26	2.6--7.5	mmol/L
前白蛋白*	0.080	↓ 0.18--0.35	g/L	肌酐*	55.5	27--66	μmol/L
丙氨酸氨基转移酶*	10.7	7--30	U/L	尿酸*	257.5	155--357	μmol/L
天门冬氨酸氨基转移酶*	36.6	↑ 8--30	U/L	β2-微球蛋白*	2.04	1.0--2.3	mg/L
谷草/谷丙	3.42			补体C3*	1.118	0.9--1.8	g/L
碱性磷酸酶*	237.5	143--406	U/L	补体C4*	0.289	0.1--0.4	g/L
γ-谷氨酰基转移酶*	<3.0	↓ 5--19	U/L	免疫球蛋白A*	0.917 ↓	1--4.2	g/L
乳酸脱氢酶*	481.5	↑ 120--250	U/L	免疫球蛋白G*	9.844	8.6--17.4	g/L
α-L-岩藻糖苷酶	9.40	≤40	U/L	免疫球蛋白M*	0.940	0.5--2.8	g/L
钾*	4.95	3.7--5.2	mmol/L	免疫球蛋白E*	21.82	≤90	IU/ml
钠*	137.9	135--145	mmol/L	降钙素原*	<0.15	<0.5无或轻度全身炎症<br>0.5-2提示中度全身炎症<br>2--10提示严重脓毒症<br>≥10 提示脓毒性休克	ng/mL
氯*	100.7	98--110	mmol/L				

实验室意见: 备注:

报告时间: 2022-11-26 09:34:58 检验者: [redacted] 审核者: [redacted]

实验室申明: 本报告仅对本次检测标本负责!
